# Regenerative medicine: today’s discoveries informing the future of medical practice

**DOI:** 10.1038/npjregenmed.2016.7

**Published:** 2016-06-09

**Authors:** Nadia Rosenthal, Stephen Badylak

**Affiliations:** 1 The Jackson Laboratory, Bar Harbor, ME, USA; 2 Imperial College London, London, UK; 3 McGowan Institute for Regenerative Medicine, Pittsburgh, PA, USA

The study of regenerative medicine has the potential to help scientists and clinicians devise early-intervention treatments for traumatic injury or degenerative diseases, by regrowth or replacement of cells or tissues. Originally an outgrowth from the field of tissue engineering and defined as the practice of replacing damaged tissues and organs or stimulating the body's own repair mechanisms to heal, regenerative medicine has now expanded to encompass the use of stem cells for modelling disease as well as autologous transplant and therapeutic delivery of functional molecules, the production of tissues and organs in a dish, the role of immune function in tissue repair, and the burgeoning area of biomedical engineering. Regenerative medicine is highly cross-disciplinary and serves as a bridge between basic science and clinical medicine.

*npj Regenerative Medicine*—a new Nature Partner Journal at the forefront of this rapidly growing research field—will enable researchers and clinicians alike to keep abreast of global developments in regenerative medicine. The open-access format of the journal means that all articles will be free to read upon publication, enabling greater reach in a timely fashion to the wider community interested in this topic, especially clinicians.

## Current challenges in regenerative medicine

A popular view is that emerging technologies disappoint in the short term and over-deliver in the long term, and it will be another 20–30 years before the full clinical benefit of regenerative medicine is realised. For the foreseeable future, much of the research in this area remains confined to the bench rather than the bedside, but clinical translation is becoming apparent.

There are many challenges in regenerative medicine, but three are noteworthy as *npj Regenerative Medicine* publishes its first articles. First, elucidation of the mechanistic aspects of embryonic development is crucial to understanding the repair of living cells and extracellular material *in situ*;^1^ however, the field is far from yielding therapeutically applicable outcomes from this knowledge.

Second, studying adult tissue turnover and replacement in an evolutionary context, using efficiently regenerating vertebrate species such as fish or salamander to analyse mechanisms of repair, often seems remote from a mammalian perspective,^2^ and relatively slow to yield mechanistic information.

Third, current approaches that rely heavily on stem cell transplantation and regenerative medicine have had minimal impact on clinical medicine so far,^3^ presumably because our understanding of the basic biology underlying tissue repair is still far from exhaustive.

These impediments notwithstanding, the potential for regenerative medicine to redress the increasing prevalence of degenerative diseases in a globally ageing population has attracted huge scientific and public interest.

## Beyond stem cells

Recent advances in our basic knowledge of the pathogenesis and histogenesis involved in tissue damage and regeneration have combined with remarkable progress in stem cell biology such that the prospect of clinical tissue repair strategies is a tangible reality.^4^ Examples include injection of stem cells or progenitor cells; induction of regeneration by biologically active molecules or inductive scaffolds administered alone or as a secretion by infused cells; immunomodulation of the scarring response; and transplantation of *in vitro* grown organs and tissues.

While there has been justifiable excitement around the use of pluripotent stem cells in regenerative medicine, how endogenous stem cells function in different tissues will need to be integrated with the biology of tissue repair. Alongside the efforts to understand the biology of transplanted cells, an equal attempt may be required to understand the recipient tissue stroma and provide the appropriate microenvironment for the transplanted cells to successfully integrate and repair tissue.^5^ The development of *in vitro* three-dimensional culture environments that prompt self-organisation of stem cells into organoids will be of particular relevance to disease modelling and treatment in the context of regenerative medicine.^6^

## The interplay between tissue stem cells and the surrounding niche

Within the context of a body, cells rarely display autonomy, but continually interact with other cells and the extracellular matrix (ECM) that surrounds and physically supports them. Studies in embryonic development and adult tissues have identified specific niches that help to control the proliferation and fate of resident stem cells. The niche is typically composed of the ECM and the resident cells, including macrophages, myofibroblasts and other immune cells. Together, these niche components help instruct and condition adult progenitor cells to promote appropriate tissue repair.^7^ Capitalising upon these events for therapeutic purposes will be particularly challenging in the setting of both acute and chronic organ injury, where signals and cellular controls underlying homeostatic replacement of healthy tissue have been distorted, rendering the patient’s body less receptive to transplanted cells in the absence of normal trophic signals.

Although a variety of possible scenarios has been proposed, effective stem cell therapy in the future will likely involve strategies for ensuring their effective function by including inductive micro-environmental niche factors with or without ECM within the target tissue to optimise survival and function of transplanted cells. Alternatively, the use of temporary niches surrounding individual transplanted cells may be required to provide appropriate survival signals. In this regard, there is an increasing recognition of a central, perhaps orchestrating role for immune cells in providing trophic signals to transplanted cells, preparing the ground for physiological tissue repair.

## Immune cellular players in tissue repair

Among the panoply of immune cells involved in the response to both acute and chronic wounds, recent findings have highlighted novel and often unexpected roles for select immune cell types in promoting a salutary environment for effective cell replacement and restoration of tissue integrity.^8^ Although cell therapy is currently a popular approach for replacing stem cells or somatic cells within a tissue, and immune suppression has been a focus of therapy, it now seems likely that immune stimulation can have positive paracrine or trophic effects upon the damaged tissue.

A rapid resolution of the pro-inflammatory phase and transition to the regeneration phase is crucial to the outcome of tissue damage, and its dysregulation may aggravate complex diseases and prevent repair. With a better understanding of the intimate relationship between immune components and affected tissues, assessment and modulation of circulating immune cells in recovering patients may provide additional clues to the extent of tissue damage and accelerate the progress of tissue regeneration.^9^ Stated differently, just as developmental biology and regenerative medicine have overlap and areas of common interest, immunology and regenerative medicine also have previously unrecognised areas of overlap with tremendous diagnostic and therapeutic potential.

## Tissue-specific features of regeneration

Effective tissue regeneration proceeds in sequential phases, including inflammation, tissue formation and maturation involving a complex orchestration of epithelial and mesenchymal cell interplay with tissue-resident and recruited immune cells. In efficiently regenerating vertebrates, this scenario plays out in a timely fashion irrespective of tissue type, whereas in mammals a remarkable disparity exists between the regenerative capacity of various tissues and organs. Thus, liver, gut, skin, kidney, skeletal muscle, heart and CNS each display unique cellular responses and signalling mechanisms in response to damage. For some organs where repair is an evolutionarily conserved feature (such as liver), insightful mechanistic experiments in other organisms can often be of relevance in mammalian systems, whereas the regenerative capacity of other organs, such as the heart or CNS, vary widely across species, making comparative studies of particular interest.

## Emerging trends in developing clinical therapies for tissue regeneration

Although our understanding of the basic mechanisms of tissue repair is still far from complete, understanding how stem cells work in context and how diseases develop during our lifespan is a crucial first step in the process of developing new treatments. The rapid clinical translation currently occurring in regenerative medicine means an increasing number of ‘first-in-human’ tissue reconstructive studies are likely, some common features of which are below:

1. The role of the immune system in tissue regeneration may provide clues for intervening in both the somatic cell and the immune response to tissue injury, by using small-molecule approaches as well as biological agents such as cytokines. As with the treatment of human autoimmune and inflammatory diseases, assessment of the molecular heterogeneity between patient immune responses in traumatic or chronic injury settings may provide vital clues to the progression of disease, and prompt design of personalised therapies.
2. Clinical tissue repair through the safe delivery of exogenous cells, be they stem cells or immune cells, entails a substantial degree of infrastructure. Implantation of composite tissues grown *ex vivo* would require a facility for cell growth and artificial biomaterials, as well as a clinical trials facility with regulatory permission, high-resolution clinical imaging and access to molecular-pathology-level tissue analysis. This multi-disciplinary environment for the clinical delivery of regenerative therapies will engage clinical research centres with a broad focus on regenerative medicine or an existing transplant program.
3. Progressive imaging of regenerating tissues and organs will be required in humans, after the stimulation of endogenous repair or the transplantation of exogenous cells, and onwards during the ageing process. Conventional imaging modalities such as MRI scanning can be used to ensure that abnormal tissue growth, such as tumours, do not form. Functional imaging that can assess tissue improvement and accompanying metabolic signatures would be ideal and would allow comparison to the original diseased and healthy ‘control’ tissue. Deviation from the healthy imaging ‘signatures’ could be understood as an early warning signal that the cells and tissues are not performing as expected, and intervention in regenerative therapy may be required. The field will also benefit from precision medical approaches to characterise individual genetic variations that may affect regenerative potential, and uncover new personalised targets for intervention.

The goal of *npj Regenerative Medicine* is to track, inform and inspire accelerated discovery in this fast-moving field to foster clinical translation. Cross-cutting studies are clearly the emerging *modus operandi*, and *npj Regenerative Medicine* will capture this new wave, encouraging multidisciplinarity, setting trends with forward-looking commentaries and spirited interchanges, and highlighting the emerging marriage of approaches, ranging from basic variation in regenerative capacity to clinical applications. Our first articles provide valuable information on therapeutic gene delivery and multigene knockout strategies, as well as novel stem cell targets for treating muscular dystrophy and a meeting review covering recent developments in the field. We are encouraged by the high quality of the content, and look forward to your future contributions!
